# Environmental assessment of Obstacle Limitation Surfaces (OLS) in airports using geographic information technologies

**DOI:** 10.1371/journal.pone.0229378

**Published:** 2020-02-26

**Authors:** Mª Rosario Contreras-Alonso, Alejandra Ezquerra-Canalejo, Enrique Pérez-Martín, Tomás R. Herrero-Tejedor, Serafín López-Cuervo Medina

**Affiliations:** 1 Departamento de Ingeniería y Gestión Forestal y Ambiental, Universidad Politécnica de Madrid, Madrid, Spain; 2 Departamento de Ingeniería Agroforestal, Universidad Politécnica de Madrid, Madrid, Spain; 3 Departamento de Ingeniería Topográfica y Cartográfica, Universidad Politécnica de Madrid, Madrid, Spain; Universidade Federal de Uberlandia, BRAZIL

## Abstract

A series of 3D obstacle limitation surfaces (OLS) define the limits to which objects may project in the airspace in order to configure the airspace around aerodromes that must be kept free from obstacles. The aim is to ensure that aircraft can safely carry out their scheduled operations, and to prevent the aerodromes from becoming unusable due to the proliferation of obstacles in the surrounding area. One such possible obstacle is the vegetation growing in the zone. This work consists of a study of the variation in the vegetation in the El Prat airport (Barcelona-El Prat Josep Tarradellas) and the surrounding area in the years 2011–2018, and of the way in which it has influenced the configuration of the OLS. Until 2010, obstacle studies were carried out every four years but the growth of plant obstacles during this period was not controlled. Although the rate of tree growth depends on several factors such as age, species, site quality and forestry treatment, the parameter analysed in this research is height, as this is what will interfere in the OLS. This study therefore focuses on measuring the height and geolocation of the obstacle in order to determine its influence on the OLS, and on determining the subsequent actions, if any, that need to be taken in regard to this vegetation element to avoid it becoming a risk to operational safety. As a result, the growth and vulnerability of 84 vegetation obstacles from 794 terrain elements have been detected. The result of this study is the design, using geographic information systems, of a tool to assist airport managers in the automated control and monitoring of the vegetation in the obstacle limitation surfaces to avoid compromising the safety of airport operations and mitigating environmental impacts.

## Introduction

Airport design standards are based on guidelines that have been developed to maintain a safe environment. A series of restrictions for buildings, installations, plantations and other elements located in the surrounding area must be established in order to guarantee the safety of the aircraft that operate in airports and aerodromes. The International Civil Aviation Organization (ICAO) defines a series of 3D OLS to ensure the safety of the scheduled operations in aerodromes and airports [1].

**Fig 1:**
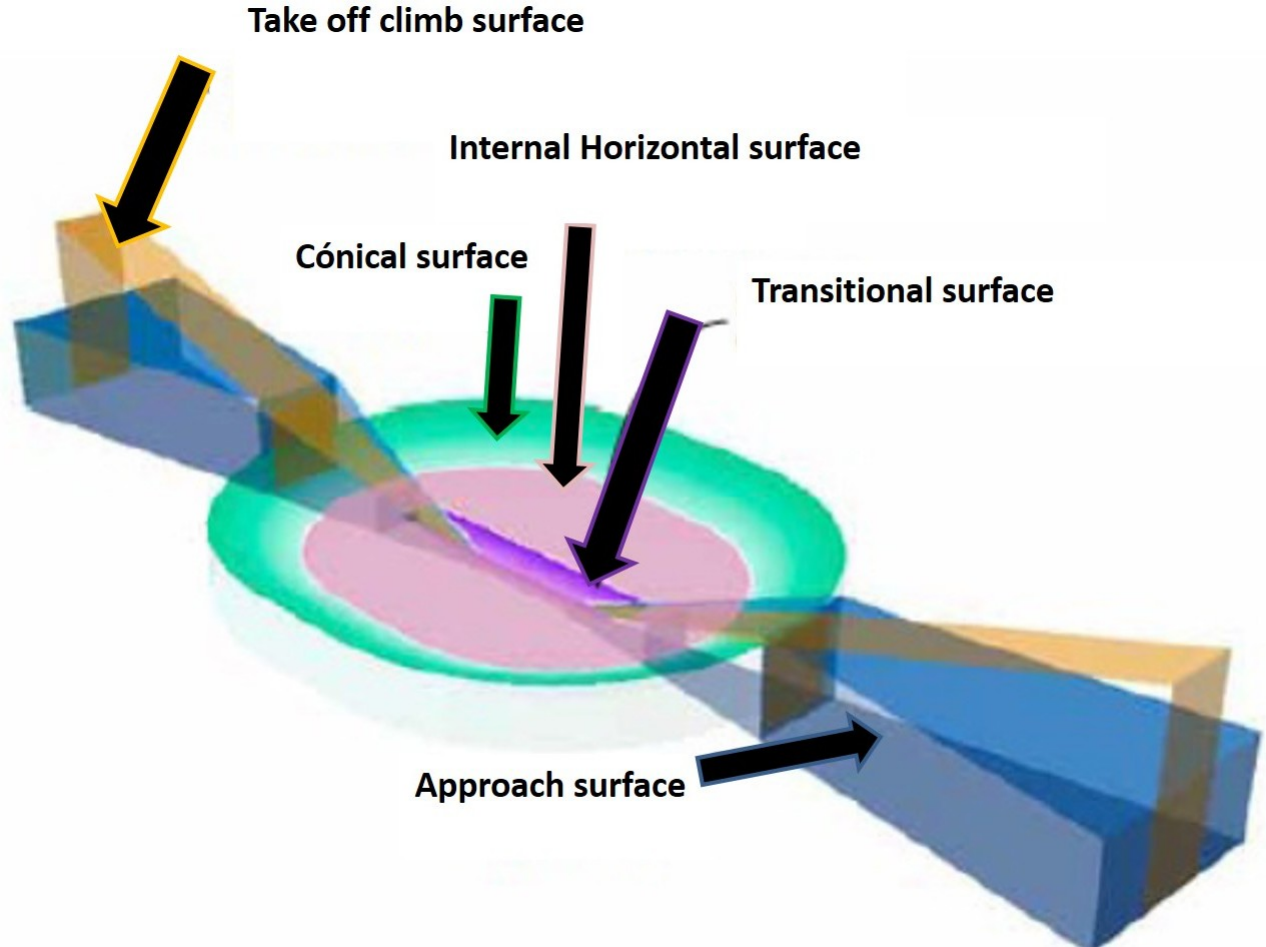
Obstacle limitation surfaces, Annex 14, aerodromes and airport services manual (doc 9137)—part 6 –limitation of obstacles.

In general terms, these limitations can be summarised as prohibiting any new obstacles from encroaching on an area around aerodromes and on their associated aeronautical radioelectric facilities. This area comprises the superimposition of a series of easements that conform the so-called aviation easement, which is established for legal effects through a legal instrument. Some studies have developed a spatial model of the airport area through the representation of a 3D cube and its smallest cells [2], while others establish imaginary 3D conical surfaces, inner horizontal surface, approach surface, inner approach surface, transitional surface, inner transitional surface, balked landing surface, and climb and takeoff surface (Fig 1). The new technologies of Geographic Information System (GIS) provide an effective and efficient mechanism in environmental modelling [3]. 3D geo-information and its utility in airport management can be analysed by integrating these obstructions in a GIS environment [4, 5].

In Spain, the approval of RD 862/2009 and the aerodrome certification process initiated by the National Air Security Agency (AESA) marked a milestone in the way airport managers deal with obstacles, by introducing the regulations and recommendations contained in Annex 14 of the International Civil Aviation Organization (ICAO) in the national legislation. An obstacle is defined by the ICAO as: “*All fixed (whether temporary or permanent) and mobile objects*, *or parts thereof*, *that are located on an area intended for the surface movement of aircraft; or extend above a defined surface intended to protect aircraft in flight*” [1].

After identifying the instances of non-compliance with the technical regulations of RD 862/2009, that is, any obstacles that extend above aeronautical easements and are not justified for operative reasons, airport management must be offered a series of solutions to eliminate or minimise these cases of non-compliance. In addition to this general framework, the El Prat airport has a further constraint: Royal Decree 2051/2004 of 11 October [6], which modifies the aviation easements of this airport in a specific way.

The obstacles are divided into two types: natural and man-made [7]. Natural obstacles include vegetation and terrain above the OLS, while man-made obstacles study objects projecting above the OLS such as posts, towers, chimneys, buildings, etc.

There are several studies conducted on obstacles related to airport operation. Hyunsu assesment the OLS and studied the prevention of bird attack risk in airport areas [8] such as natural obstacle study. Others have studied the man-made obstacles impact in their studies as Kim that evaluated the rules and criterias to ensure the safety of aircraft operating around the airport [9] or DoHyun, who established obstacle limitation criteria for specific airport conditions [10]. The present study focuses particularly on vegetation elements, mainly trees, which are living beings whose dimensions change over time and must be specifically controlled to ensure they do not project above the OLS.

It should be noted that the area of application of the proposed methodology has the particularity that it is a protected area. This implies that the actions needed to maintain the OLS clear of obstacles are restricted by the regulations, and require a very clear justification. The aim is therefore to design a procedure that allows the rigorous control of the growth (height) of the vegetation elements and minimises the necessary actions without compromising the safety of airport operations, while causing the least possible damage to the environment.

Prado and collaborators develop a methodology to generate the obstacle drawings of five airports in Mexico from digital aerial images obtained with small format cameras [11]. The traditional method for geolocating obstacles is to use topographical techniques supported by GPS receivers [12]. Due to the absence of specific maps of the airport area, and the difficulty and high cost of observations owing to the size of the area and the number of obstacles, studies have tended to focus on implementing new techniques based on surface modelling [13] [14]. The three-dimensional terrain model has been generated in studies using LiDAR techniques, supported by aerial photographs and satellite images [15] [16, 17], obtaining a precision of 20 cm horizontally and 10 cm vertically. Vertical precisions of 3 to 10 m have been achieved thanks to interferometric techniques with radars located on space platforms and information from high-resolution satellite images [18]. Photogrammetric techniques with digital area images obtained with a small format camera have also been used [19]. Cuadra studies the installation of wind farms and their impact on the OLS of airport spaces [20]. In addition to establishing the OLS by capturing geo-referenced data, some studies have collected real data on flight trajectories using the information supplied by air traffic control radars [21].

Due to new mass data acquisition techniques, digital surface models (DSM) as opposed to digital terrain models (DTM) have in recent years been used to detect the heights of the various obstacles and compare their evolution over time [22]. It is essential to ensure that aircraft are not at risk of collision as a result of the presence of buildings, trees, aerials, lamp posts, cranes and other elements that do not comply with height restrictions and are located within the airport safety surfaces, so the maps of the obstacles on the aerodrome must be constantly updated to guarantee the safety of the air operations.

Previously to this study, only obstacle checks were carried out every four years, which is a risk because there are no vegetation growth data refered to OLS during that period. The present work aims to develop a current methodology for identifying, managing and controlling the vegetation obstacles in the OLS in the El Prat airport. The proposed methodology allows to minimize the environmental actions guaranteeing the conservation of the natural resources.

## Material and methods

### Study area

**Fig 2:**
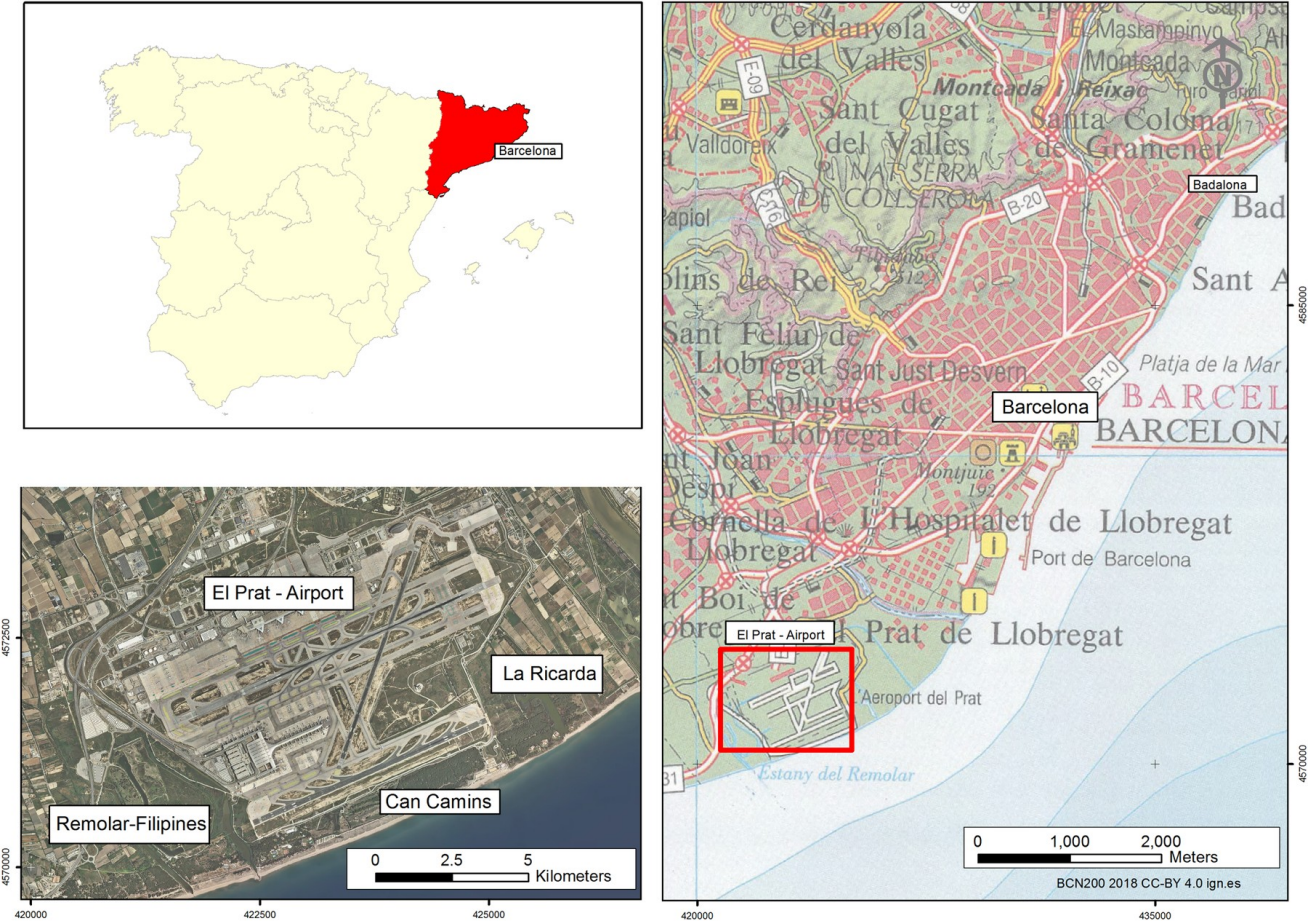
Plan of the situation and special protection areas (SPA) in the natura 2000. BCN200 2018 CC-BY 4.0 ign.es.

The Barcelona city airport (Spain) is in the southwest of the metropolitan area and has various specific physical and environmental characteristics (Fig 2). All this environment is naturalised and protected, and the space surrounding the Braç de la Vidala and the Estany del Remolar has become a marsh, the Marisma de las Filipinas (part of the special protection area for birds (SPA) (Natura 2000 network). As this is a protected area, any actions on the species (pruning, removing stems…) are severely restricted, so the measuring process must be extremely rigorous and precise in order to justify these studies.

The definition, situation and dimensions of the areas and surfaces that configure the easements and their slopes all depend on the runway code letter. This code letter is assigned according to the runway’s basic length, which is obtained by applying a series of corrections by height, slope and temperature. The aim is to describe the imaginary surfaces according to Annex 14 –Aerodromes and Airport Services Manual (Doc 9137)—Part 6 –Limitation of obstacles, so they can be controlled and monitored; and to identify the obstacles so they can be notified and/or published in the AIP (Aerodrome obstacle plan–OACI, Type A, B and C (Aeronautical Charts Manual–Doc 8697-AN889/2)). El Prat airport has three runways: 07L/25R, 07R/25L and 02/20, with the OLS main characteristics shown in Table 1.

**Table 1:** Description of the OLS in the El Prat airport with details of the maximum elevations and slopes in each OLS in the airport.

**TAKE OFF CLIMB SURFACE**	**07L**	**25R**	**07R**	**25L**	**20**	**2**
*Divergence (%)*	12.5	12.5	12.5	12.5	12.5	
*Distance between the inner and the outer edge*	15000	15000	15000	15000	15000	
*Climb/Ascent slope (%)*	2	2	2	2	2	
*Elevation of the inner edge (m*.*)*	2.94	2.41	2.42	2.44	2.03	
*Elevation of the outer edge (m*.*)*	302.94	302.41	302.42	302.44	302.03	
**APPROACH**	**07L**	**25R**	**07R**	**25L**	**20**	**2**
*Divergence (%)*	15					
*Distance between the inner and the outer edge*	15000					
*First segment*	*Starts at the inner edge*					
*Elevation (m*.*)*	2.47	2.94	2.44	2.42		2.03
*Second segment*	*Starts at 3000 meters from the inner edge*					
*Elevation (m*.*)*	62.47	62.94	62.44	62.42		62.03
*Slope (%)*	2.5	2.5	2.5	2.5		2.5
*Maximum elevation (m*.*)*	254.48	152.94	152.44	152.42		152
*Third segment*	*Starts at 6660 meters from the inner edge*					
*Elevation (m*.*)*	152.44	152.94	152.44	152.42		152
**TRANSITION**						
*Slope (%)*	14.3					
Departs from the elevation of the corresponding runway strip and from the approah surfaces, until reaching 49.32m above the internal horizontal surface						
**INTERNAL HORIZONTAL SURFACE**						
Horizontal plane located at a height of 49.32 meters (45 meters above the ARP) defined by the envelope of all the circles centered on the reference points of each runway and with a radius of 4000m						
**CONICAL SURFACE**						
*Elevation of the inner edge (m*.*)*	49.32					
*Slope (for 2000 m) (%)*	5					
*Maximum elevation (m*.*)*	149.32					
**OBSTACLE PROTECTION**	**07L**	**25R**	**07R**	**25L**	**20**	**2**
**SURFACE PAPI**						
*Divergence (%)*	15	15	15	15		15
*Distance between the inner and the outer edge*	15000	15000	15000	15000		15000
*Slope (%)*	3.14	3.37	3.52	3.22		3.22
*Elevation of the outer edge (m*.*)*	473.47	508.44	530.44	485.42		485

The safety height varies depending on the distance to the runway, so the permitted heights are very low in the area closest to it and, increase the greater the distance away from it. The viability of the vegetation formations inside the aforementioned area is conditioned by their height, which directly affects the pine stands in Can Camins, La Ricarda, the military zone, El Remolar and a small riverbank copse within this last area.

### Data and data collection

The study took place from January 2011 to May 2018, using data supplied by the El Prat Airport from the “*report on the determination of obstacles in the approach*, *takeoff*, *transition*, *OFZ (obstacle-free zone)*, *inner horizontal*, *conical and circuit areas at the El Prat Airport”* in October 2010 (AENA 2010). This information was supplied and processed on a spreadsheet and paper documentation, highlighting the need to integrate all data into a GIS.

Two categories of geo-information can be distinguished:

- Maps of the terrain and all the artificial elements in the environment of the El Prat Airport generated from the obstacle determination reports, the 1/5000 scale map, satellite images, GNSS measuring campaigns and classic topography.
- The obstacle limitation surfaces (OLS) created according to the descriptions and measurements in Annex 14 and the Airport Services Manual.

Once the possible obstacles and sensitive areas were identified, a topographic measuring campaign was carried out to determine the X, Y, Z coordinates of all the possible vegetation obstacles and elements to be controlled in order to identify and quantify all those that extend above an OLS and any others less than 10 m below.

The project reference bases were determined and positioned using the Global Navigation Satellite System (GNSS) working in differential kinematic mode in real time through the Internet/General Packet Radio Service (GPRS) with the Networked Transport of RTCM protocol via Internet Protocol (NTRIP), and Virtual Reality Solutions (VRS) technology, and the closest station with baselines of less than 15 km. The network used was the National Geodesic Network of GNSS reference stations of the National Geographic Institute (ERGNSS). The topographic monitoring bases were thus provided with UTM ETRS89 ZONE 31 coordinates. In regard to coordinate Z, a specific geoid model was created for all bases located within the airport premises with the orthometric heights of the vertices of the airport's own airport control network (RCTA), as this produces a model that corresponds more closely to the actual form of the terrain in this area. The official Spanish geoid model EGM2008-REDNAP was used for the bases located outside, as they do not have any known points of orthometric height.

**Fig 3:**
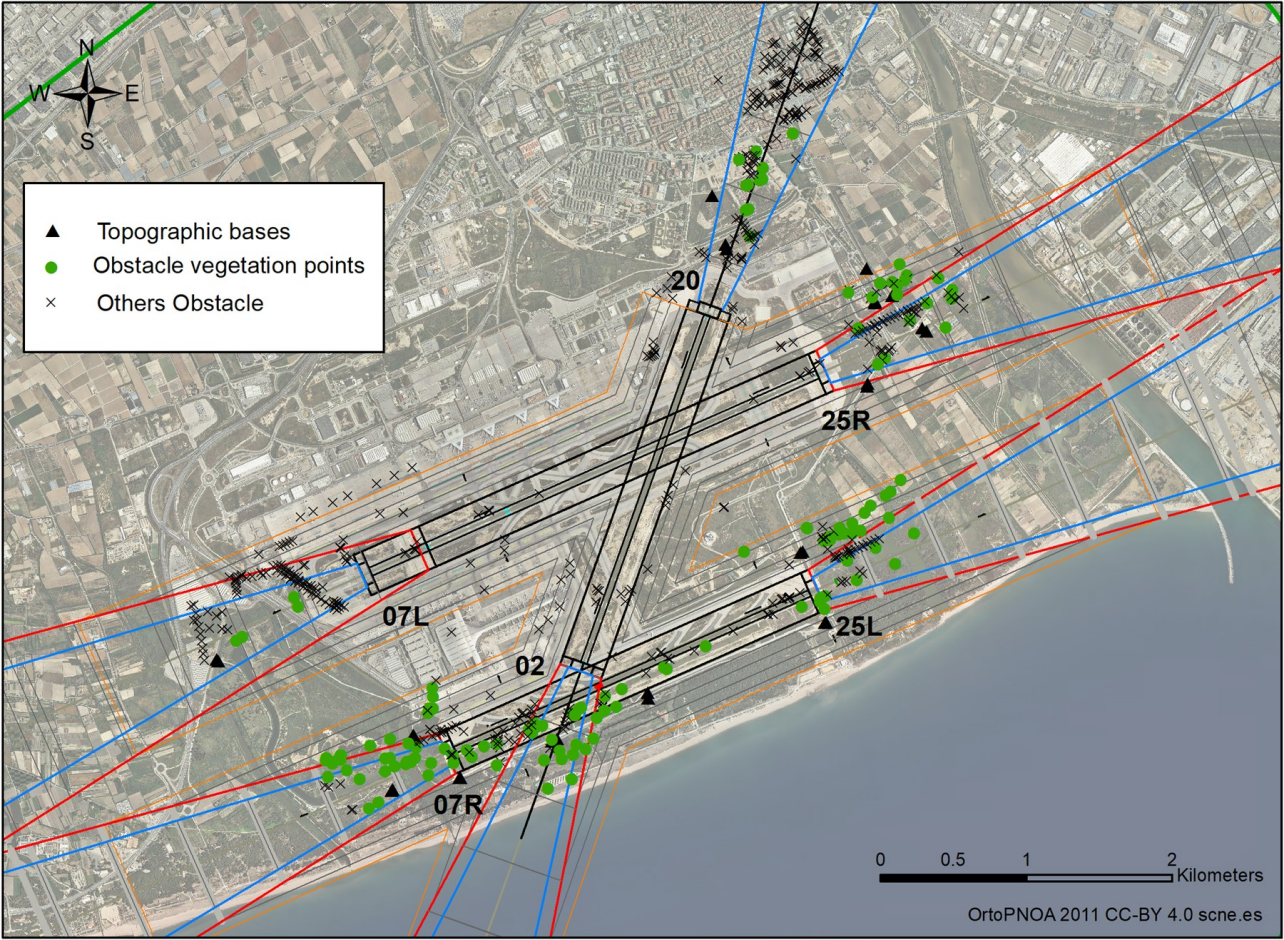
Topographic bases and vegetation obstacles and elements for monitoring. CC-BY 4.0 scne.es 2011.

The bases took the form of feno markers if they were located on natural terrain, or steel-type spikes on asphalt, thus guaranteeing their permanence during the research study. A total of 26 topographic bases were established and installed, and these constitute the basis for the measurements for monitoring and controlling the vegetation obstacles and other elements (Fig 3).

The first measurement campaign was conducted after calculating the X, Y, Z coordinates of the project reference bases in 2011. The topographic radiation method was used with a Topcon GPT 7500 total measuring station in non-prism mode. Data were taken from 1302 vegetation elements, of which 84 were obstacles and 394 elements to control. The highest branch of each vegetation element was visualised from each base, always taking care to record the measurements in meteorological conditions of little wind to avoid distorting the measurements [23].

### Data modelling and analysis

Before starting the modelling data and analysis, the information supplied by El Prat Airport was verified, completed and modified to adapt it to the study objective. The decision was made to create two versions, one in ETRS89 UTM zone 31 and another in geographic coordinates WGS84. The origin of the altitudes in both versions was established as the average level above the sea in Alicante, as this is the origin of altitudes in aeronautics and in the Spanish territory.

The GIS comprises five images from the Geoeye satellite, commissioned exclusively for this study and with a frequency of three months from 2011 to 2013, and six months from 2013 to 2017. Images from the Geoeye-1 satellite were previously oriented using control points from GNSS and classic topographic field compaigns. The orthorectification was made by the Erdas Multipoint Geometric Correction module in which each pixel of the MDE is taken and associated with its equivalent position in the image and an orthomosaic re-scaled to 8 bits for its visualisation.

The mapped information (IC) was then modelled, generating a Digital Terrain Model (DTM) through a triangulated irregular network (TIN), as this allows a more real representation of the obstacles and elements to be monitored.

**Fig 4:**
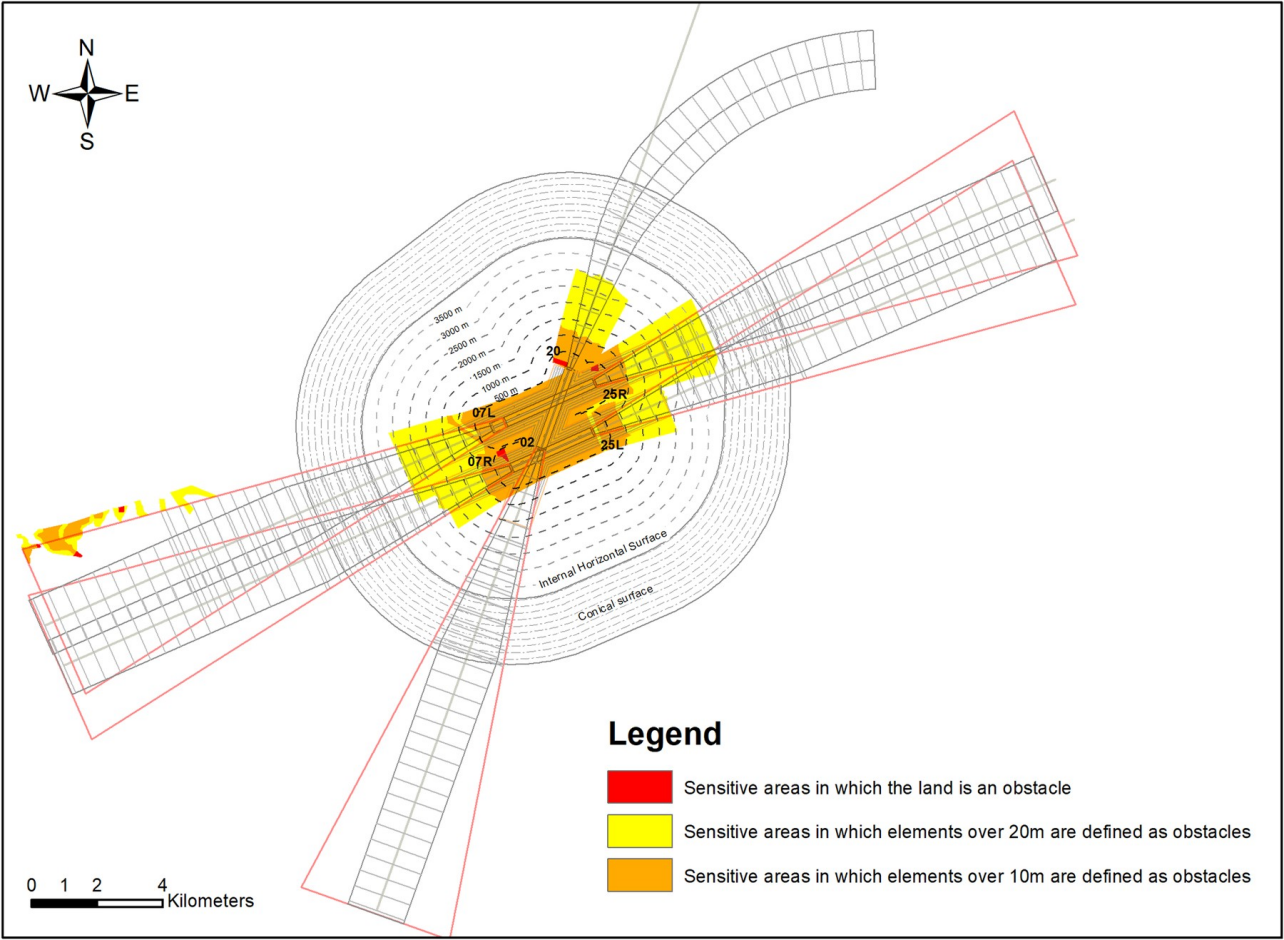
Sensitive areas classified by colours according to the distance at which they would become an obstacle.

The OLS were also modelled from 3D polylines, generating a TIN surface. The vegetation obstacles were identified and analysed with the AutoCAD Civil 3D software by geoprocessing the areas in the MDT that extended above an OLS, and the sensitive areas where any vegetation element whose height exceeded the distance would represent an obstacle in each case (Fig 4). We also identified the vegetation elements to be controlled, with the difference that the TIN surface representing the OLS was replaced by a TIN surface in which the OLS height was 10 m lower.

An application was implemented in the CAD environment (HAOSS) in which both the OLS and the possible obstacles and elements to be controlled are automatically represented by 3D solids, so that when the solids in the OLS intersect with those of each possible vegetation obstacle or element, the altimetric distance between them is automatically quantified. This generates a database with the coordinates of the obstacles and elements and the positive or negative height according to whether or not they extend above the OLS.

These data were used to analyse which parameters or attributes needed to be determined for each obstacle or element and to create the geodatabase. This was done by consulting the flight field personnel at the El Prat Airport in order to incorporate the attributes that they considered relevant.

**Fig 5:**
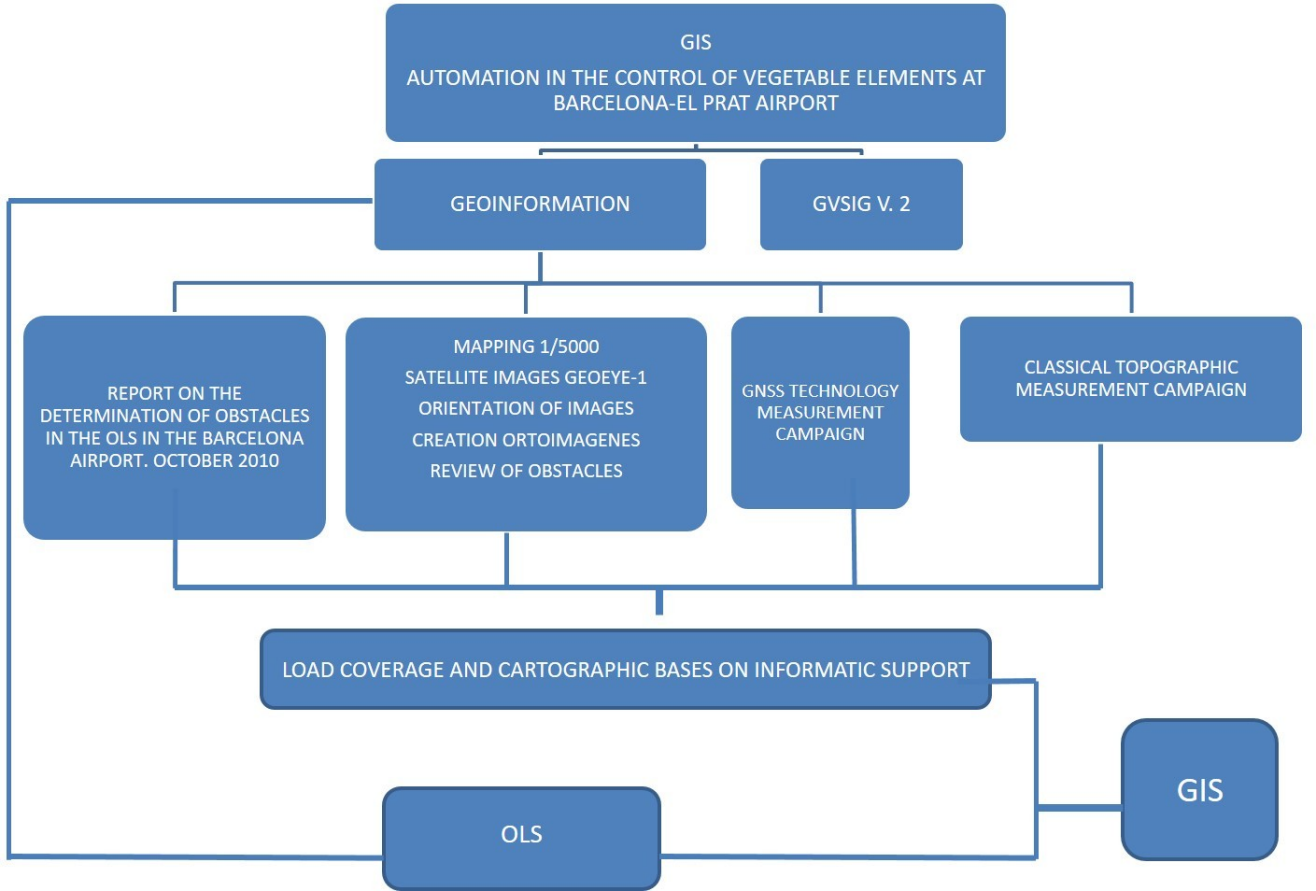
Diagram of GIS components.

The supplied data, their modifications and new captured information were combined into a new GIS model that allow new control and monitor processing following the next diagram (Fig 5).

## Results

**Fig 6:**
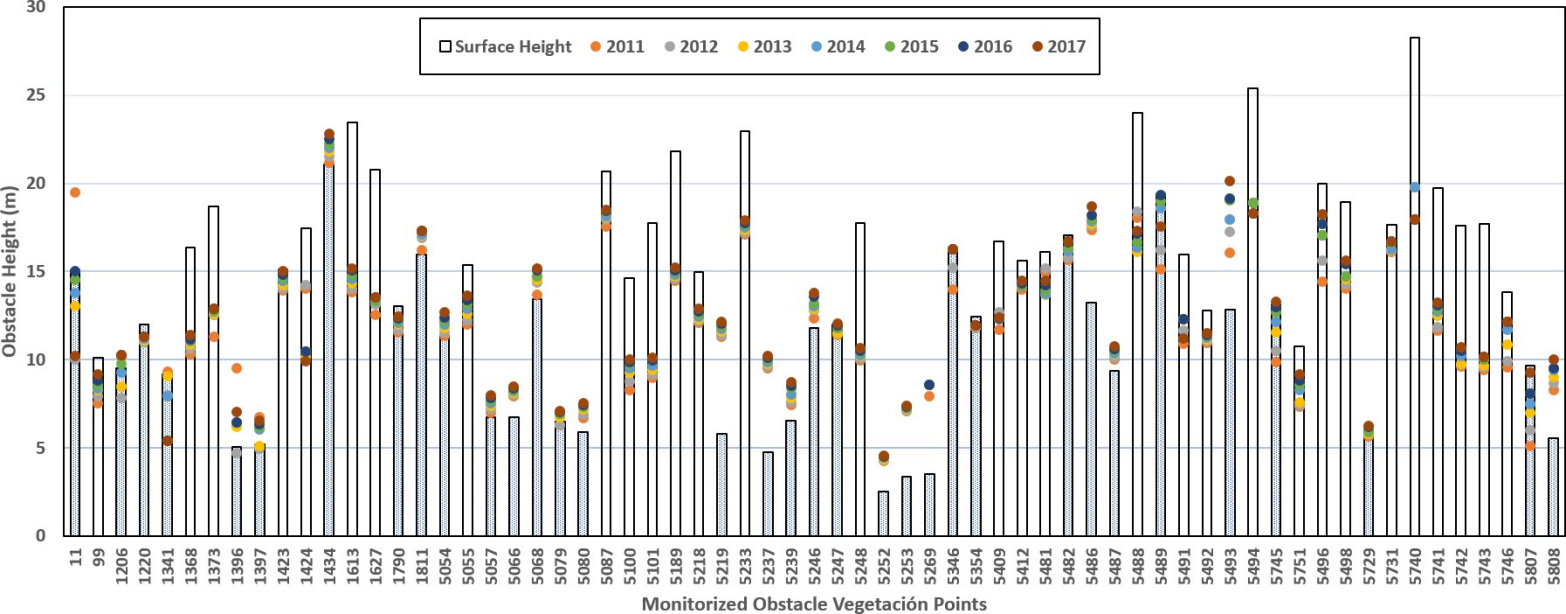
Growth and applied actions in vegetation obstacle. Shadow bars represent obstacles that requires priority actions. Dispersion points show the year growth values.

As results of the proposed methodology, the study determines the type of geometry to represent each vegetation obstacle and element for monitoring, as there are tree formations in the study area (airport and area around the El Prat Airport) in which the trees are very closely spaced, making it more appropriate to consider them as extensive elements and represent them by means of polygons. All this was integrated and organised as shown in the following table: 84 vegetation obstacles (6.46%) and 384 obstacle elements (30.30%) from 1302 obstacle elements requiring monitoring were detected, a result that justifies the usefulness of this study, as the characteristics of the environment around the El Prat Airport mean that vegetation elements are among the most abundant, important and yet changeable types of obstacles and elements for monitoring, representing 35% of the all obstacles. This thus confirms the importance of monitoring and controlling them closely (Fig 6).

**Fig 7:**
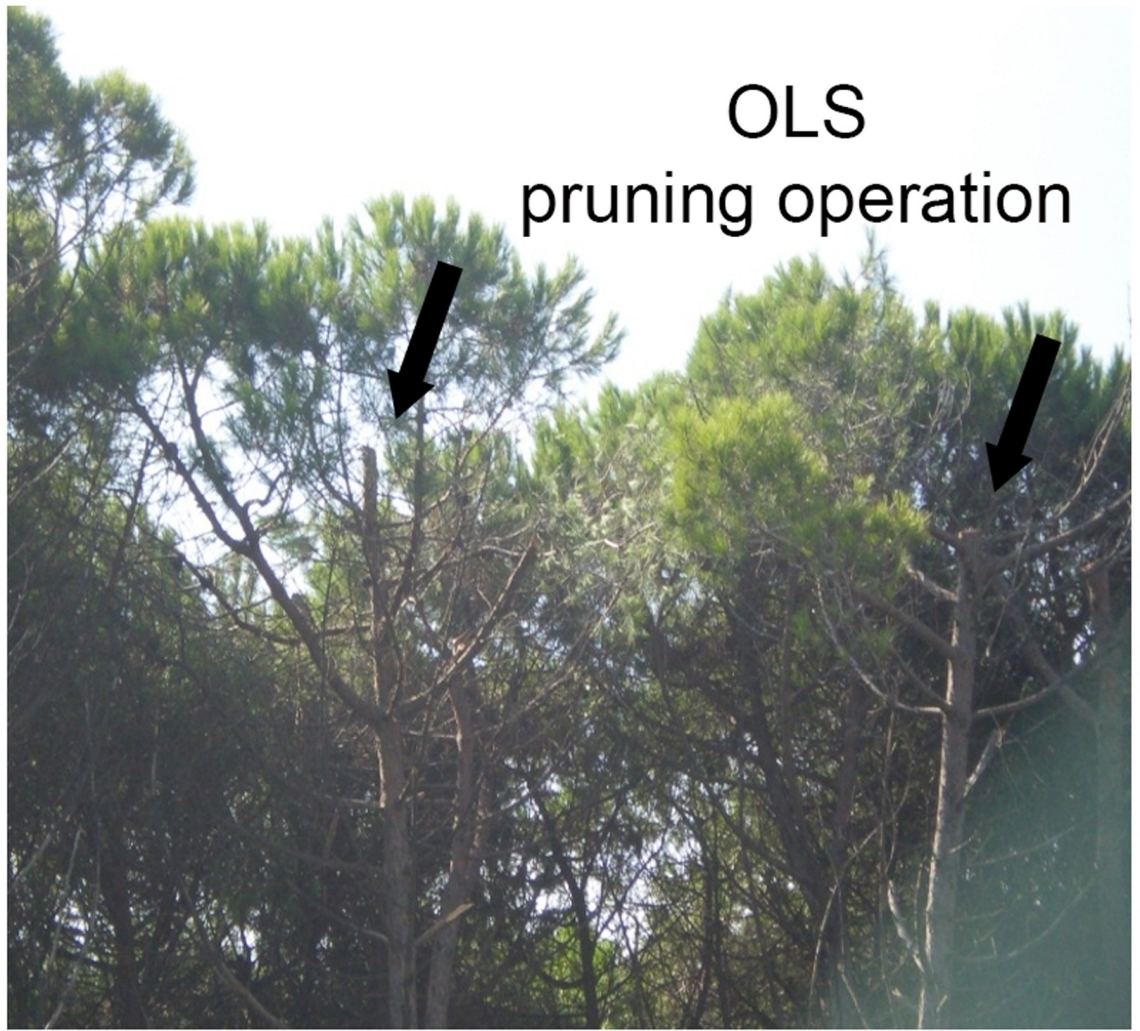
Photograph showing the pruning of two pines previously considered to be isolated elements, and for which–because they were within a wooded area–the proposed pruning operation resolved the infringement of the OLS in the medium term.

Maintenance actions such as pruning or cutting establish negative growth meanwhile positive quantities show the year growth variations (Fig 7). Table 2 shows the quantity values that obstacles have to be cutted during the pruning process.

**Table 2:** OLS vulnerability database. https://doi.org/10.3886/E115661V1.

Point	Inside-Outside	Specie	Altitude	OLS 1	Altitude	Vulnerated	OLS 2	Altitude	Vulnerated
					OLS1	Height 1		OLS2	Height 2
11	Outside	Ulmus minor	19.49	Transitional 07L-25R	15.05	4.43	-	-	-
99	Outside	Pinus pinea	9.15	Approach 25R	10.10	-0.38	-	-	-
1206	Outside	Pinus pinea	10.28	Approach 25L	9.53	3.63	Take off climb 07R	9.53	3.63
1220	Outside	Ulmus minor	11.29	Approach 25L	12.01	-0.72	-	-	-
1341	Outside	Arecaceae	9.34	Transitional 07R-25L	9.16	0.18	-	-	-
1368	Outside	Arecaceae	11.39	Approach 02	16.37	-4.98	-	-	-
1373	Outside	Arecaceae	12.88	Approach 02	18.71	-5.83	-	-	-
1396	Inside	Pinus pinea	9.52	Transitional 07R-25L	5.06	4.45	-	-	-
1397	Inside	Pinus pinea	6.75	Approach 07R	5.19	1.55	-	-	-
1423	Outside	Ulmus minor	14.81	Without affection	-	-	-	-	-
4080	Outside	Pinus pinea	149.55	Conical	135.44	14.11	-	-	-
5237	Outside	Ulmus minor	10.02	Approach 25L	4.77	5.25	PAPI 25L	6.20	3.82
5239	Outside	Arecaceae	7.60	Approach 25L	6.54	1.06	Take off climb 07R	6.54	1.06
5246	Outside	Pinus pinea	12.42	Transitional 07R-25L	11.78	0.64	-	-	-
5252	Outside	Tamarindus	4.55	Approach 25L	2.51	2.04	PAPI 25L	2.57	1.98
5253	Outside	Tamarindus	7.37	Approach 25L	3.39	3.97	PAPI 25L	3.99	3.38
5269	Outside	Tamarindus	8.11	Approach 25L	3.51	4.60	PAPI 25L	4.18	-

**Fig 8:**
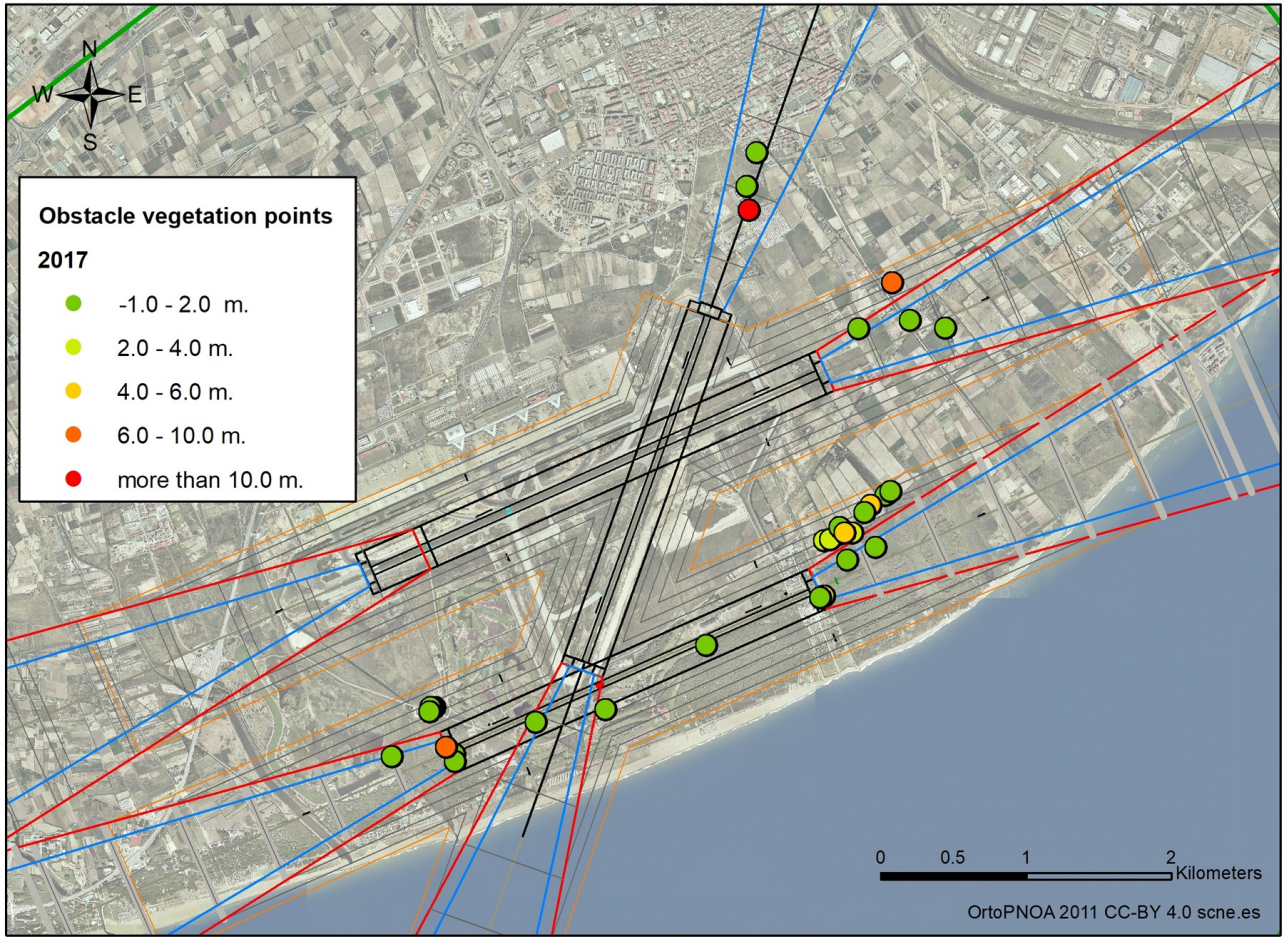
Vulnerability values of vegetation obstacle in 2017. CC-BY 4.0 scne.es 2011.

The use of geographic information systems allows keeping information updated. Locate the position of all vegetation obstacle related to other elements and show the vulnerabilities height for planning maintenance. (Fig 8).

## Discussion

The implementation of the GIS allowed us to obtain the growth statistics, determine potentially hazardous elements and take preventive decisions, among other results, which was the aim of this work. It was therefore decided to schedule the monitoring and control operations on a quarterly basis based on the results of Fig 6 keeping the elevation of the obstacles inside the vertical boxes.

Improved definition of vegetation elements, as their visualisation on the satellite images allowed the identification of the elements that form vegetation stands and of others that were previously considered to be isolated vegetation elements, leading in the past to totally ineffectual pruning operations (see Fig 7) that acted on an isolated element rather than resolving the problem.

The growth of vegetation elements depends on their species, on the conditions in the site where they are located and on the forestry conditions to which they are subjected. As can be deduced from the data analysed by the GIS, all the common *pinea* specimens located in protected spaces present a greater height development than those in poorer areas.

The application allows the identification of all the vegetation obstacles that belonged to AENA (Aeropuertos Españoles y Navegación Aérea–Spanish Airports and Aerial Navigation) in order to propose a pruning regime and determine the scope of the pruning to ensure they remain one metre below the corresponding OLS.

A GIS Based methodology tool allows quickly oversee of the natural obstacle maintenance thanks to their know position and monitoring capability that reduce to a check review those elements in shorts periods of time.

## Conclusions

The methodology developed solves the difficulties of the airport manager for the monitoring and control of obstacles and plant elements located in the OLS. The result allows minimizing the actions to be carried out in order to preserve the restrictions of the protected environment.

The methodology of this study is being used as a reference in actions in environmental impact studies in other Spanish airports. Satellite images, maps, MDE, SLO, measurement data and significant attributes or parameters are implemented in a single application, thus allowing analyses and assessments reduces costs by eliminating the ineffectual pruning operations of the past, and making the actions more precise and efficients.

The GIS based approach for assessing Obstacle Limitation Surfaces (OLS) in airports solves the determination of vegetation elements and automates the detection of potential hazars as necessary preventive measures for the reduction of vegetation obstacles and any elements that are likely to become an obstacle.

Detection of areas whose conditions are better suited to the growth of trees, possibly due to the favourable conditions of the environment, as is the case of the protected spaces of El Remolar-Filipinas, La Ricarda, etc. These require more frequent control, so quarterly reviews are proposed. Similarly, certain zones with slower tree growth have been detected which therefore require less frequent controls, allowing twice yearly reviews. This has led to cost reductions and an optimisation of the use of resources that corresponds more closely to the real need.

It is open as a new line of research to develop the application of new registration tools such as mobile mapping system capable of precise determining vegetable elements or obstacles and allowing to verify their growth and data integration into GIS.

## Supporting information

S1 Data (XLSX)
